# *In vivo* Treg expansion under costimulation blockade targets early rejection and improves long‐term outcome

**DOI:** 10.1111/ajt.16724

**Published:** 2021-08-23

**Authors:** Christoph Schwarz, Benedikt Mahr, Moritz Muckenhuber, Anna Marianne Weijler, Lukas Walter Unger, Nina Pilat, Michaela Latus, Heinz Regele, Thomas Wekerle

**Affiliations:** ^1^ Section of Transplantation Immunology Division of Transplantation Department of General Surgery Medical University Vienna Vienna Austria; ^2^ Division of Visceral Surgery Department of General Surgery Medical University Vienna Vienna Austria; ^3^ Clinical Institute of Pathology Medical University Vienna Vienna Austria

**Keywords:** basic (laboratory) research/science, costimulation, immunosuppressant ‐ other, immunosuppression/immune modulation, T cell biology

## Abstract

CTLA4Ig has been shown to improve kidney allograft function, but an increased frequency of early rejection episodes poses a major obstacle for more widespread clinical use. The deleterious effect of CTLA4Ig on Treg numbers provides a possible explanation for graft injury. Therefore, we aimed at improving CTLA4Ig's efficacy by therapeutically increasing the number of Tregs. Murine cardiac allograft transplantation (BALB/c  to B6) was performed under CTLA4Ig therapy modeled after the clinically approved dosing regimen and Tregs were transferred early or late after transplant. Neither early nor late Treg transfer prolonged allograft survival. Transferred Tregs were traceable in various lymphoid compartments but only modestly increased overall Treg numbers. Next, we augmented Treg numbers *in vivo* by means of IL2 complexes. A short course of IL2/anti‐IL2‐complexes administered before transplantation reversed the CTLA4Ig‐mediated decline in Tregs. Of note, the addition of IL2/anti‐IL2‐complexes to CTLA4Ig therapy substantially prolonged heart allograft survival and significantly improved graft histology on day 100. The depletion of Tregs abrogated this effect and resulted in a significantly diminished allograft survival. The increase in Treg numbers upon IL2 treatment was associated with a decreased expression of B7 on dendritic cells. These results demonstrate that therapy with IL2 complexes improves the efficacy of CTLA4Ig by counterbalancing its unfavorable effect on Tregs.

## Abstract 2

In vivo expansion of Tregs by IL‐2 complexes acts synergistically with CTLA4Ig therapy to down regulate available B7 on antigen‐presenting cells.

**Abbreviations**
APC: antigen‐presenting cell
CNI: calcineurin inhibitor
Cplxs: complexes
CTLA4: cytotoxic T lymphocyte‐associated protein 4
GILS: graft infiltrating lymphocytes
MST: median survival time
SD: standard deviation
Treg: T regulatory cells

## 1 INTRODUCTION

B7‐targeted costimulation blockade with cytotoxic T lymphocyte‐associated protein 4 (CTLA4)Ig provides an attractive alternative to common immunosuppressive therapy with nephrotoxic calcineurin inhibitors (CNIs). The beneficial effects on kidney function and long‐term patient/graft survival have been established ^1^, ^2^, ^3^, ^4^ while early rejection episodes still remain a major concern.^5^ Therefore, new strategies are warranted to improve CTLA4Ig‐based regimens by preventing early rejection. CTLA4Ig inhibits signaling through CD28 which impairs intrathymic regulatory T cell (Treg) generation^6^ and the survival of peripheral Tregs. In particular, CTLA4Ig impairs peripheral Treg homeostasis directly by blocking the crucial survival signals through CD28 and indirectly by prohibiting the production of IL2 by conventional CD4 T cells. IL2 is a prerequisite to maintain peripheral Treg numbers and plays a decisive role in Treg expansion and activation.^7^, ^8^, ^9^, ^10^ Tregs are particularly important to maintain self‐tolerance^11^ but also play a pivotal role for solid organ transplantation.^12^ It is hypothesized that the reduced number of Tregs facilitates the occurrence of early rejection episodes,^13^ but causal proof is lacking in the clinical setting and other factors might also contribute to belatacept‐resistant rejection.

In line with this, we could recently show that Tregs rapidly decline under chronic CTLA4Ig therapy but slowly return thereafter to baseline levels.^14^ The extent and course of Treg reduction was independent of the administered dose while efficacy with regard to organ survival appeared to be dose dependent. In fact, BALB/c heart allografts were gradually rejected under low doses (10 mg/kg BW, i.e., the dose approved for clinical use) of chronic CTLA4Ig therapy but survived indefinitely if high and very high doses (five‐ or 25‐fold higher dose) were administered. Moreover, the efficacy of low‐dose CTLA4Ig was further impaired if Tregs were depleted. Accordingly, we hypothesized that therapeutically increasing Treg numbers at the time of transplantation would improve heart allograft survival under low‐dose CTLA4Ig. Two strategies to increase recipient Treg numbers were investigated—the adoptive transfer of Tregs and the pharmacological *in vivo* expansion by means of IL2/aIL2 complexes (IL2 cplxs).

## 2 MATERIAL AND METHODS

All experiments were approved by the local review board of the Medical University of Vienna and the Austrian Federal Ministry of Science, Research and Economy and were performed in accordance with national and international guidelines of laboratory animal care. Female BALB/c (H‐2^d^) and C57BL/6 (H‐2^b^) mice were purchased from Charles River Laboratories (Sulzfeld) and were housed under specific pathogen‐free conditions.

### 2.1 Cardiac transplantation

Heterotopic cardiac transplantation was performed as previously described.^15^ In brief, the recipient's external jugular vein (EJV) and common carotid artery (CCA) were everted over a cuff under the microscope. Donor heart harvesting included injection of 1 ml of heparin solution in the inferior vena cava. After opening the thorax, the heart was flushed with 4 ml of HTK solution (Custodiol, Koehler Chemie) through the aortic arch. After careful resection from the thorax, the pulmonary trunk was connected with the EJV and the aortic trunk with the CCA. Graft survival was assessed by visual inspection and palpation daily immediately posttransplant and at least twice weekly during long‐term follow‐up. Rejection was defined as complete cessation of heartbeat and was confirmed in histological analysis.

### 2.2 Antibody treatment

Mice were treated with 0.25 mg (≈10 mg/kg BW) hCTLA4Ig (Abatacept; Bristol‐Myers, Squibb Pharmaceuticals [Princeton, NJ].) administered on D0, 4, 14, 28, and then every 4 weeks (low‐dose regimen).^14^ This control group of CTLA4Ig monotherapy (*n* = 7) was pooled with mice treated with the same regimen that have already been shown in previous work (*n* = 9).^14^ In groups of mice, Treg depletion was performed with anti‐CD25 mAb (PC61; 0.25 mg) on days −1 and 3. Treg depletion was confirmed by flow cytometry of peripheral blood on day 7 following transplantation using a non‐crossreacting anti‐CD25 mAb (7D4).

### 2.3 Treg transfer

In vitro‐activated Tregs were generated as described previously.^16^ In short, CD4^+^ CD25^+^ cells were separated from the spleen and lymph nodes of C57BL/6 mice by magnetic bead separation (MACS; Miltenyi Biotech). CD4 cells were first enriched by withdrawing undesired cell populations. CD4‐enriched cells were then positively selected for CD25. CD4^+^ CD25^+^‐separated Tregs were cultured for 5 days in 12‐well plates in RPMI‐1640 media (Biochrome) supplemented with 200 U/ml IL2 (Sigma‐Aldrich), 10% fetal calf serum (Linaris), PenStrep (100 U penicillin, 100 g streptomycin per milliliter; Sigma‐Aldrich), 10 mM HEPES (MP Biomedicals), 1 mM sodium pyruvate (Sigma‐Aldrich), 1x nonessential amino acids (Sigma‐Aldrich), and 10 μM b‐mercaptoethanol (Sigma‐Aldrich). The plates were precoated with 10 mg/ml of a‐CD3 (145‐2C11; Bio X Cell) and 1 mg/ml of a‐CD28 (37.51; BioLegend) in phosphate‐buffered saline overnight at 4°C. At the time of transfer, 90% of CD4^+^ CD25^+^ T cells expressed Foxp3.

### 2.4 IL2 cplxs

IL2 cplxs were prepared by incubating 1 μg of recombinant mouse IL2 (eBioscience) with 5 μg of purified a‐mouse IL2 (clone JES6‐1A12) (BioXcell) for 30 min at 37°C. IL2 cplxs were administered i.p. in a final volume of 300 μl. Selected groups of mice were treated with IL2 cplxs on D‐3, D‐2, and D‐1 prior to heart transplantation.

### 2.5 Flow cytometric analysis

For analysis of immune cell subsets, cells were stained with CD3‐PerCp‐Cy5 (17A2), CD4‐APC‐Cy7 (RM4‐5), CD25‐PE‐Cy7 (PC61), CD45.1‐BIO (A20), CD45.2‐BIO (104), CD80‐FITC (16‐10A1), CD11c‐PE (N418), and FoxP3‐APC (FJK‐16s). Intracellular staining was performed with a FoxP3 staining kit (eBioscience) as previously described.^17^ Flow cytometry was performed on BD FACS Canto II (BD Biosciences), and data were analyzed by FlowJo (10.0.8) software. Graft‐infiltrating lymphocytes (GILS) were measured after digestion of the cardiac allograft using a mouse tumor dissociation kit (Miltenyi). Briefly, cardiac allografts were harvested 14 days post‐transplant, cut in small pieces, and incubated for 40 min at 37°C for enzymatic digestion. After lysis of remaining red blood cells, the resulting single cell suspension was prepared for flow cytometric analysis as described above.

### 2.6 Mixed lymphocyte reaction

Mixed lymphocyte reaction was adapted from Tourkova et al.^18^ In brief, dendritic cells were isolated on day 14 after cardiac transplantation by magnetically activated cell sorting (Milteny). T cells were isolated from naïve C57BL/6 splenocytes using the “Pan T cell isolation kit, mouse” from Miltenyi. Separated T cells (C57BL/6; 4 × 10^5^) and 4 × 10^5^ dendritic cell depleted and irradiated (30 Gy) BALB/c (donor) PBMCs were incubated in triplicates with or without the addition of isolated dendritic cells (in a ratio of 1:10) for 5 days. For measurement of proliferation, responder T cells were stained with VPD.

The percentage of proliferation of T cells incubated with BALB/c in combination with dendritic cells was normalized on the percentage of proliferation of T cells incubated with BALB/c alone for calculation of stimulation indices (SI).

### 2.7 Histological analysis

Mice were sacrificed and grafts were harvested at the time of rejection, at 2 weeks or 100 days after transplantation, as indicated. Samples were fixed in 7.5% formalin overnight, embedded in paraffin, and subsequently sectioned and stained with hematoxylin and eosin. Grading was performed according to the International Society for Heart and Lung Transplantation (ISHLT) guidelines for cellular rejection score by a blinded pathologist.

### 2.8 Statistical methods

Statistical analysis was performed with GraphPad Prism 5 (GraphPad Software Inc.). Ordinal variables were compared with a Fisher's exact test or a chi‐squared test. Metric values were shown as mean with standard deviation (SD) or median, and statistics was performed with an unpaired *t*‐test or with a Mann‐Whitney *U* test. Survival curves were estimated according to the Kaplan‐Meier method and compared to each other with a log rank test. A *p*‐value <.05 was considered statistically significant.

## 3 RESULTS

### 3.1 Costimulation blockade has no major impact on the survival of transferred Tregs

**FIGURE 1:**
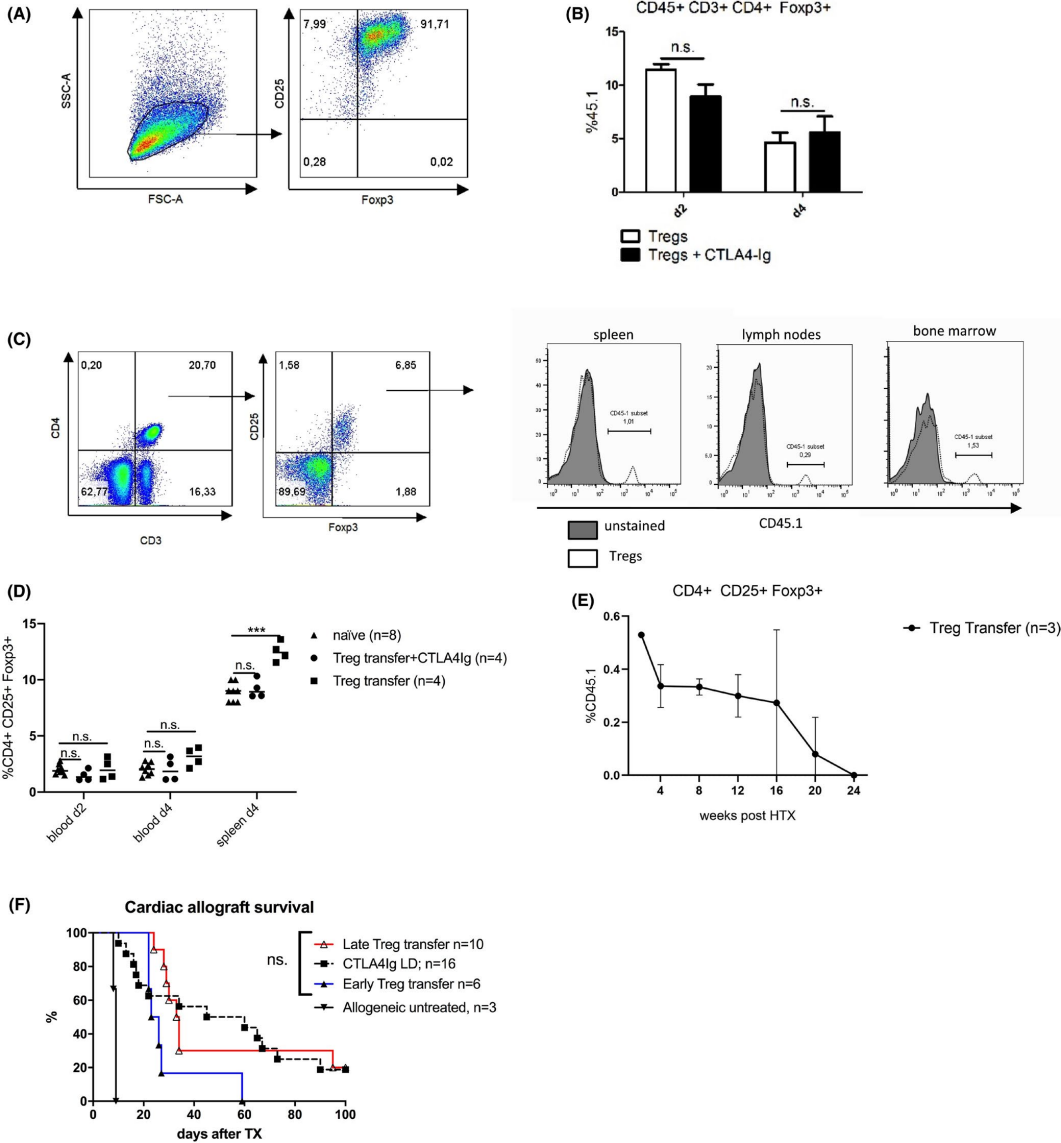
Adoptive Treg transfer and costimulation blockade: 3 × 10^6^ CD45.1 Tregs were adoptively transferred (D‐1) to CD45.2 naïve BL6 mice treated with or without (w/o) a single injection of CTLA4Ig (D0). (A) The cell product contained more than 90% of CD25+ Foxp3+ Tregs. (B) The amount of CD45.1^+^ cells among CD4^+^ CD25^+^ Foxp3^+^ Tregs was analyzed 3 (D2) and 5 (D4) days posttransfer in the blood. (C) Five (D4) days posttransfer, CD45.1 Tregs could be recovered in the spleen, lymph nodes, and bone marrow of CTLA4Ig‐treated recipients. (D) A significant but only modest increase in total Treg numbers was observed in the spleen. This increase was absent in the event of CTLA4Ig therapy. No significant difference could be observed in the blood, independent of CTLA4Ig therapy. (E) The transferred Tregs were traceable in the blood, albeit to a small extent, more than 16 weeks after transplantation. (F) However, neither early nor late adoptive Treg transfer resulted in prolonged allograft survival [Color figure can be viewed at wileyonlinelibrary.com]

At first, we aimed to increase Treg numbers by adoptive transfer of in vitro‐activated Tregs. To test whether costimulation blockade affects the survival of transferred Tregs, 3 × 10^6^ CD45.1 Tregs were transferred (D‐1) into naïve congenic CD45.2 recipients treated with (*n* = 4) or without (*n* = 4) CTLA4Ig (0.25 mg, D0). The injected cell product contained more than 90% of CD25^+^ Foxp3^+^ Tregs (Figure 1A). CTLA4Ig did not adversely affect the numbers of transferred Tregs in the blood as comparable amounts of CD45.1 cells could be recovered among CD4^+^ CD25^+^ Foxp3^+^ Tregs 3 and 5 days posttransfer (Figure 1B). Five days posttransfer, transferred Tregs were detected in different tissues including the spleen, lymph nodes, and bone marrow (Figure 1C). However, in spite of the relatively high number of transferred Tregs, a significant, but only modest, increase in overall Treg frequencies was observed in the spleen of naïve congenic CD45.2 recipients. This increase was absent in the event of CTLA4Ig therapy as CTLA4Ig reduces the number of endogenous Tregs.^14^ No differences could be observed in the blood, independent of CTLA4Ig therapy (Figure 1D). These data suggest that CTLA4Ig does not substantially affect the survival of adoptively transferred Tregs.

### 3.2 Treg transfer does not improve the cardiac allograft survival under costimulation blockade

To test whether transferred Tregs prolong heart allograft survival under costimulation blockade, we transferred 3 × 10^6^ Tregs 1 day before (early) or 8 days after (late) transplantation. The transferred Tregs were detectable in the blood, albeit to a small extent, up to 16 weeks after heart transplantation (Figure 1E). However, neither early nor late adoptive Treg transfer improved the heart allograft survival under costimulation blockade (Figure 1F). Thus, it appears that the adoptive transfer of Tregs was not sufficiently potent to compensate for the decrease in Treg numbers caused by CTLA4Ig.

### 3.3 IL2 cplxs restore Treg numbers under CTLA4Ig

**FIGURE 2:**
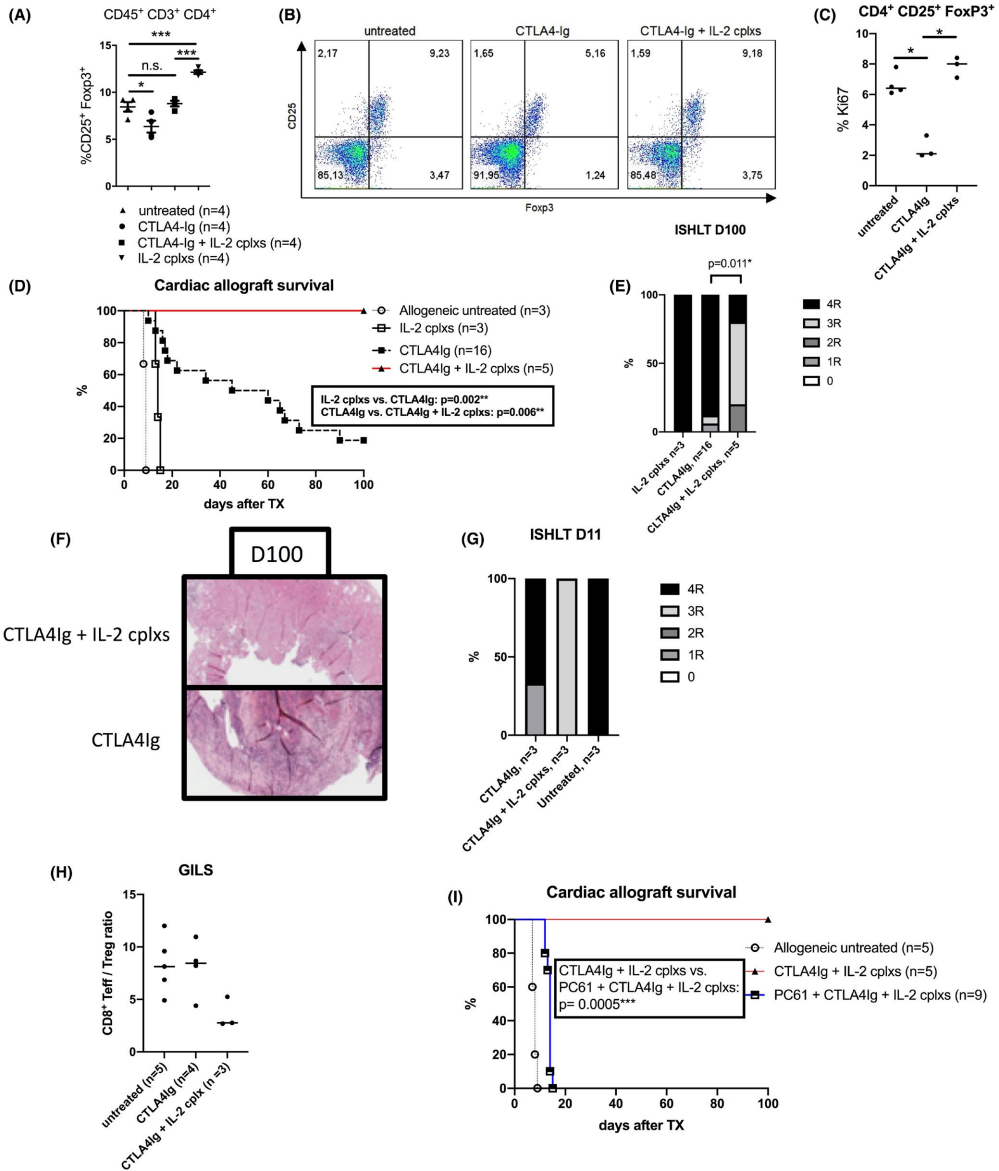
IL2 cplxs and CTLA4Ig: naïve C57BL/6 mice received either CTLA4Ig (*n* = 4), IL2 cplxs (*n* = 4), or a combination of both (*n* = 4). (A, B) CD4^+^ CD25^+^ Foxp3^+^ Tregs were compared in the spleen 9 days after CTLA4Ig administration. Two‐color dot plots show representative mice. (C) Ki67 expression in CD4^+^ CD25^+^ Foxp3^+^ Tregs in the spleen 9 days after CTLA4Ig administration. (D) C57BL/6 recipients of BALB/c hearts were treated with chronic CTLA4Ig and a short course of IL2 cplxs on 3 consecutive days (d‐3, d‐2, and d‐1) before transplantation. Heart allograft survival was significantly prolonged when combining CTLA4Ig with IL2 cplxs (*n* = 5) compared to CTLA4Ig monotherapy (*n* = 16) (*p* = .006). (E) Histology on day 100 or the time of rejection revealed a significantly improved grade of rejection when combining costimulation blockade and IL2 cplxs compared to monotherapy. (F) Representative image of hearts retrieved on day 100 after transplantation. (G) When analyzing early rejection, we observed a trend toward a lower grade of allograft rejection with IL2 cplxs on day 11 after transplantation while the heart was still beating (*n* = 3). (H) Graft infiltrating lymphocytes were analyzed with flow cytometry on day 14 following transplantation. There was a trend toward a more favorable (i.e., lower) CD8 T eff/Treg ratio in mice under CTLA4Ig + IL2 cplx therapy (*n* = 3) compared to CTLA4Ig monotherapy (*n* = 4) and untreated controls (*n* = 5). (I) Treg depletion, performed on days −1 and 3 (*n* = 9), led to a drastically diminished allograft survival compared to mice under CTLA4Ig and IL2 cplx therapy without Treg depletion (*n* = 5) (*p* = .0005) [Color figure can be viewed at wileyonlinelibrary.com]

As an alternative approach, we expanded Tregs therapeutically *in vivo* by means of IL2 cplxs. Complexing IL2 with a specific antibody (clone JES6‐1A12) against IL2 (anti‐IL2) has been shown to selectively expand Tregs.^19^ As expected, three consecutive doses of IL2 cplxs (1 μg/5 μg) induced a rapid expansion of Tregs in blood 2 days after the last dose. This increase was significantly higher than the one following adoptive Treg transfer (Figure 2A).^19^, ^20^

To test whether IL2 cplxs restore Treg numbers under CTLA4Ig, groups of mice were treated with a single dose (D0) of CTLA4Ig (0.25 mg) with or without IL2 cplxs (D‐4, D‐3, D‐2). As shown previously, Treg numbers significantly dropped upon CTLA4Ig treatment compared to naïve mice.^14^ The proliferation marker Ki67 was significantly reduced in Tregs after CTLA4Ig treatment implying that their reduced numbers resulted from a decreased rate of proliferation. Adding IL2 cplxs to CTLA4Ig treatment restored Treg numbers to baseline values by stimulating their proliferation (Figure 2B,C). Treg numbers after IL2 cplx treatment were significantly lower with CTLA4Ig treatment than without, indicating that CTLA4Ig mitigates the effects of IL2 cplxs. Notably, IL2 cplxs restored peripheral Treg numbers after CTLA4Ig treatment by promoting their proliferation.

### 3.4 IL2 cplxs improve allograft survival under costimulation blockade

To test the impact of IL2 cplxs on cardiac allograft survival, groups of mice under CTLA4Ig therapy were treated with or without IL2 cplxs before transplantation (D‐3, D‐2, D‐1). Mice receiving only chronic CTLA4Ig therapy rejected their allograft within a median survival time (MST) of 52.5 days. Notably, adding IL2 cplxs to CTLA4Ig therapy significantly improved graft survival with all grafts surviving until the end of the observation period (=100 days) (*p* = .006). All mice receiving only IL2 cplxs without CTLA4Ig therapy rejected their allograft within 14 days (IL2 cplxs vs. CTLA4Ig LD: 14 days vs. 52.5 days; *p* = .002) (Figure 2D). Histological analysis on day 100 or the day of rejection revealed a significantly better preserved graft histology in mice with combined therapy compared to CTLA4Ig‐treated mice (*p* = .011) (Figure 2E,F). Thus, IL2 cplxs improve the efficacy of CTLA4Ig therapy preventing immune‐mediated graft loss.

### 3.5 IL2 cplxs ameliorate early graft rejection

To investigate the underlying mechanisms of IL2/aIL2‐mediated improvement of allograft outcome, we transplanted mice under CTLA4Ig with or without IL2 cplxs and early graft injury was assessed by histological inspection 2 weeks posttransplant (*n* = 4). In mice with combined costimulation blockade and aIL2/IL2 therapy, we observed a trend toward reduced allograft rejection scores compared to CTLA4Ig and to untreated naïve controls, respectively (Figure 2G). Analyzing graft infiltrating lymphocytes via flow cytometry revealed higher numbers of Tregs and lower numbers of CD8^+^ effector T cells resulting in a reduced CD8 eff T cell/Treg ratio (Figure 2H).

### 3.6 Cardiac allograft survival under CTLA4Ig + IL2 cplx therapy is Treg‐dependent

To assess whether Tregs play a pivotal role for allograft survival under costimulation blockade and IL2 complex therapy, we depleted Tregs by using an anti‐CD25 mAb on day −1 and day +3 (*n* = 9). Treg depletion was confirmed on day 7 via flow cytometry by using a non‐crossreacting anti‐CD25 mAb (clone 7D4) (data not shown). Upon Treg depletion, allograft survival was abrogated compared to mice without Treg depletion (MST = 14 days vs. >100 days; *p* = .0005) indicating a causal role for Tregs in the graft‐prolonging effect of IL2 cplx therapy under CTLA4Ig (Figure 2I).

### 3.7 IL2 cplxs decrease CD80 expression on dendritic cells

Finally, we aimed to gain more insight by which mechanism IL2 cplxs were able to reduce the necessary dose of CTLA4Ig for infinite heart allograft survival. We speculated that the reduced number of Tregs under CTLA4Ig therapy might increase the availability of B7 (CD80, CD86) expressed on antigen‐presenting cells (APCs) as Tregs steadily remove B7 molecules from APCs via CTLA4 by a process termed transendocytosis.^21^ Thus, we measured B7 expression on dendritic cells in the spleen 9 days after a single injection of CTLA4Ig. CD80 levels significantly increased upon treatment with CTLA4Ig, probably as a consequence of decreased Treg numbers while CD86 levels remained unchanged (Figure S1A–C). Notably, adding IL2 cplxs restored CD80 levels to values observed in untreated mice.

**FIGURE 3:**
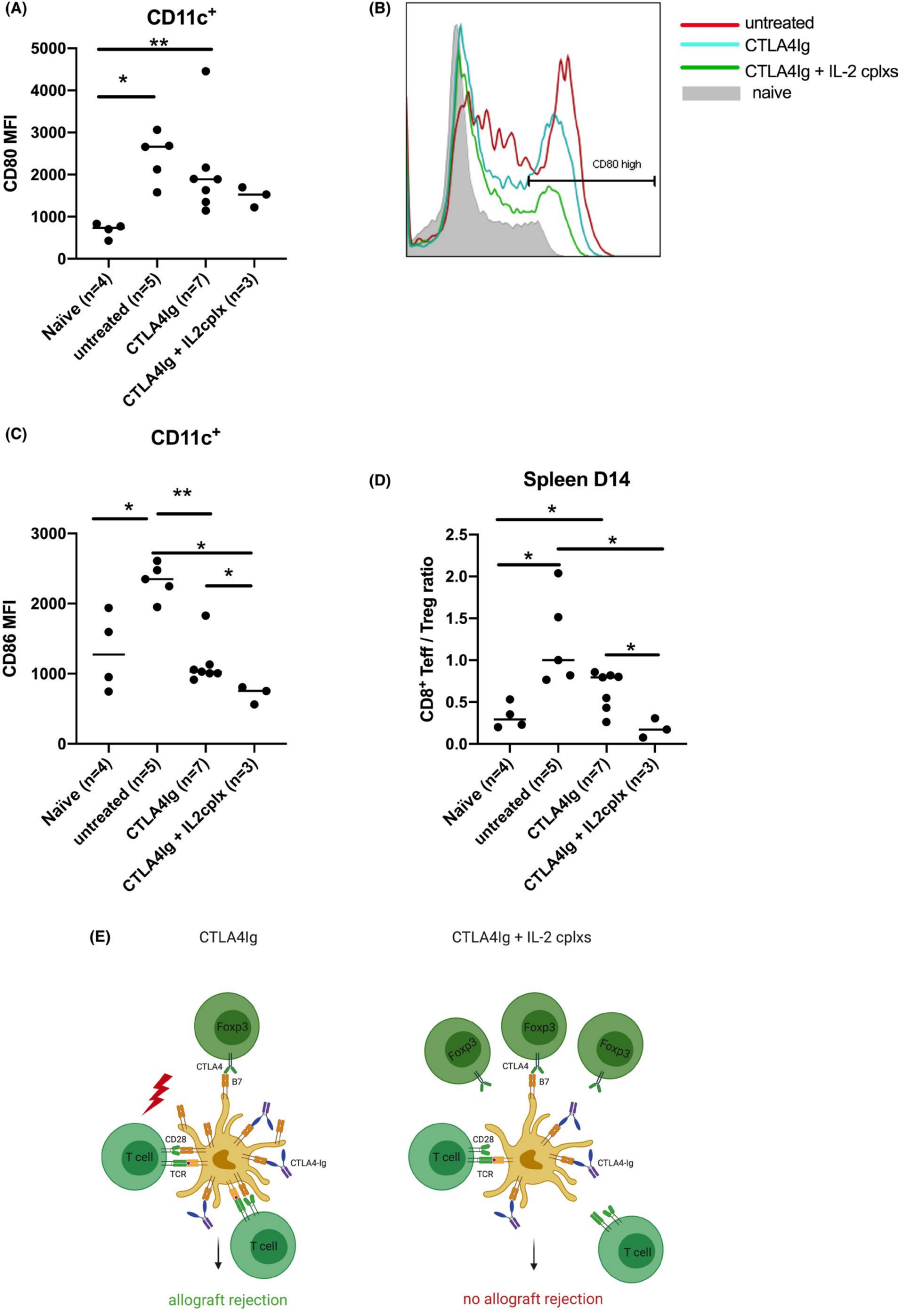
Mechanisms of IL2 cplx treatment—B7 (CD80, CD86) expression was measured on dendritic cells in the spleen 14 days after cardiac transplantation under various treatment regimens. (A, B) CD80 expression levels significantly increased in untreated mice upon allograft rejection (*n* = 5) and in mice under CTLA4Ig monotherapy (*n* = 7), but less so in mice treated with CTLA4Ig plus IL2 cplxs (*n* = 3). (C) Similarly, CD86 expression was significantly increased in untreated mice compared to naïve controls (*n* = 4). CTLA4Ig + IL2 cplx (*n* = 3)‐treated mice showed significantly lower CD86 levels compared to mice under CTLA4Ig monotherapy. (D) CTLA4Ig and IL2 cplx treatment significantly decreased the CD8 T eff/Treg ratio compared to untreated controls and CTLA4Ig monotherapy, respectively. (E) Schematic of potential mechanism of action: results suggest that the mechanism of action includes an increased number of Tregs by the aIL2/IL2 therapy that results in reduced expression of B7 molecules on antigen‐presenting cells that can be controlled/ligated by low‐dose costimulation blockade [Color figure can be viewed at wileyonlinelibrary.com]

Next we measured CD80 and CD86 on CD11c^+^ dendritic cells in mice after cardiac transplantation. On day 14 following transplantation, CD80 and CD86 were significantly increased in untreated mice upon allograft rejection compared to naïve mice (Figure 3A–C). While CTLA4Ig prevented the upregulation of CD86 on dendritic cells observed in untreated recipients following transplantation, its effect on CD80 surface expression on DCs was limited. Importantly, the addition of IL2 cplxs further reduced the surface expression of CD80 and CD86 on dendritic cells compared to CTLA4Ig monotherapy (Figure 3C). An unfavorable CD8 T eff/Treg ratio was noted in untreated controls and CTLA4Ig LD‐treated mice. However, the combination of CTLA4Ig and IL2 cplxs led to an increase in Tregs in combination with reduced numbers of CD8 T eff cells which resulted in a significantly improved CD8 T eff/Treg ratio (Figure 3D). Notably, CD8 Teff cells were reduced to the levels observed in naïve mice (without transplant) in heart transplant recipients treated with CTLA4Ig + IL2 cplx.

In a next step we wanted to test whether dendritic cells from mice after cardiac transplantation with different treatment regimens have distinct stimulatory capacities given the varying expression of B7 molecules. Dendritic cells were harvested on day 14 following transplantation by magnetically activated cell sorting (MACS, Miltenyi). Naïve C57BL/6 T cells were stimulated with DC‐depleted, irradiated BALB/c splenocytes with and without the addition of dendritic cells from sensitized C57BL/6 cardiac allograft recipients. Proliferation was measured with VPD staining via flow cytometry. We observed a trend toward an increased T cell proliferation in the presence of dendritic cells from untreated mice and lower rates of T cell responses in the presence of dendritic cells from mice under CTLA4Ig with or without IL2 cplxs (Figure S1D).

In summary, we conclude that CTLA4Ig indirectly increases the expression of B7 molecules on APCs by reducing the number of Tregs which otherwise constantly remove B7 molecules. The increased amount of B7 molecules in turn requires a higher dose of CTLA4Ig to prevent heart transplant rejection. IL2 cplxs restore the physiological number of Tregs by promoting their proliferation which in further consequence reduces the number of B7 molecules. As a result, a lower CTLA4Ig dose, such as the one used in clinical routine, is sufficient to achieve permanent heart graft survival (Figure 3D).

## 4 DISCUSSION

Herein we show for the first time a synergistical therapeutic effect of IL2/aIL2 complexes with CTLA4Ig that allows long‐term allograft survival in a stringent murine transplant model. We suggest that the increased heart allograft survival can be attributed to the increased number of Tregs upon IL2/aIL2 treatment, which—among other effects—restores physiological expression levels of B7 on dendritic cells. Further, we establish that CTLA4Ig can be safely combined with Treg cell therapy.

Early allograft rejections are still a major concern in belatacept‐based immunosuppression. Even though the overall outcome does not seem to be affected by these rejection episodes, this remains an obstacle for more widespread use. We have shown previously that the immunosuppressive capacity is at least partly dependent on the presence of Tregs.^14^ As the negative impact of CTLA4Ig on Treg numbers is well established, ^14^ we aimed at increasing the number of Tregs in order to improve allograft outcome. Transferred Tregs can suppress the proliferation of directly alloreactive T cells in the draining lymph nodes after heart transplantation. Since directly alloreactive T cells start to proliferate 4 days after transplantation, we decided to assess the number of Tregs 5 days posttransfer.^22^ There was no major negative impact on the survival of transferred Tregs under CTLA4Ig therapy. The transferred Tregs were traceable up to 16 weeks following transplantation. However, neither early nor late polyclonal Treg transfer resulted in a positive impact on allograft survival. One explanation might be that the number of Tregs was not sufficiently increased, even though a Treg dose was transferred that was higher than those used in clinical trials so far (i.e., 12 × 10*7/kg)^23^. Since peripheral Treg are highly dependent on IL2, it seems possible that transferred and endogenous Tregs compete for existing IL2 and thereby limit the overall increase in Treg numbers.

We have previously shown that the immunosuppressive capacity of CTLA4Ig is Treg‐dependent at low but not high doses. There are at least three distinct explanations for these observations: first, there might be an augmented alloreactivity of the T cell repertoire under Treg depletion. Second, there could be synergistical effects of Tregs and CTLA4Ig which are underlined by the similar mechanism of action (decreasing biological available B7 molecules on APCs by transendocytosis [Tregs] or binding [CTLA4]). Activated effector T cells also transiently express CTLA4 and thereby also contribute to the reduction of costimulatory molecules, but Foxp3 Tregs are likely to play a particularly important role here due to their constitutive expression.^24^ A third option is that the number of Tregs is known to be connected to the number of available B7 molecules expressed on the surface of APCs. It is known that, as Tregs “rip‐off” B7 molecules,^21^ the presence of Tregs decreases B7 on APCs. Conversely, a decrease in the number of Tregs results in an upregulation of B7 on APCs. Thus, in the case of CTLA4Ig, Treg numbers are reduced under costimulation blockade, with the result of a higher B7 expression on APCs. As low‐dose CTLA4Ig under Treg depletion (e.g., fewer Tregs, more B7) results in a deleterious transplant outcome and higher doses of CTLA4Ig resolve the negative impact under Treg depletion, we hypothesize that under low‐dose therapy, there are free and therefore biologically available B7 molecules that are only saturated under high‐dose therapy.

Besides the Treg‐induced effect on B7 regulation, it is well known that T eff cells might activate APCs, thus the reduced expression of B7 on dendritic cells might also be an indirect effect caused by a reduced T cell response. By administering IL2 cplxs, we could increase the number of Tregs in mice under CTLA4Ig therapy to numbers observed in untreated animals. IL2 complexes probably compensate for the decreased production of endogenous IL2 under CTLA4Ig therapy.^25^ Moreover, the increased expression of B7 molecules on APCs under costimulation blockade was significantly lowered. Together this resulted in indefinite survival in all mice. Most strikingly, the combination of CTLA4Ig + IL2 cplxs led to a favorable impact on Treg numbers not only systemically but also within the graft. The observed effect of *in vivo* Treg expansion was Treg‐dependent as Treg depletion almost completely prevented the graft prolongation seen with CTLA4Ig + IL2 cplxs without Treg depletion. This explanation does of course not rule out additional mechanisms by which Tregs suppress alloreactivity and promote graft acceptance.^26^

One advantage of the proposed combination therapy is that IL2 is already clinically available and was shown to selectively increase Treg numbers in humans.^27^, ^28^ Therefore, the combination of IL2 and CTLA4Ig might be a clinically feasible approach to target early allograft rejection in transplant patients. The administration of IL2 in the clinic is challenging due to its short half‐life and its severe side effects (e.g., vascular leak syndrome) at higher doses.^25^ However, IL2 has recently been shown to improve clinical outcome in chronic graft versus host disease in steroid‐refractory patients. Sixty‐one percent showed a partial response by week 12 and 30% had a stable disease. Response predictors were a shorter time between the onset of chronic graft versus host disease and treatment with IL2 and a Treg:Tcon ratio of more than 0.7.^29^

We conclude that the addition of IL2 cplxs improves allograft outcome under costimulation blockade with CTLA4Ig. The potential clinical efficacy of this therapy needs to be determined in future clinical trials.

## DISCLOSURE

The authors of this manuscript have no conflicts of interest to disclose as described by the *American Journal of Transplantation*.

## Supporting information

Fig S1

## Data Availability

Data available on request from the authors.

## References

[1] Vincenti F , Rostaing L , Grinyo J , et al. Belatacept and long‐term outcomes in kidney transplantation. N Engl J Med. 2016;374(4):333‐343. 26816011 10.1056/NEJMoa1506027

[2] Vincenti F , Larsen C , Durrbach A , et al. Costimulation blockade with belatacept in renal transplantation. N Engl J Med. 2005;353(8):770‐781. 16120857 10.1056/NEJMoa050085

[3] Durrbach A , Pestana JM , Pearson T , et al. A phase III study of belatacept versus cyclosporine in kidney transplants from extended criteria donors (BENEFIT‐EXT study). Am J Transplant. 2010;10(3):547‐557. 20415898 10.1111/j.1600-6143.2010.03016.x

[4] Vincenti F , Charpentier B , Vanrenterghem Y , et al. A phase III study of belatacept‐based immunosuppression regimens versus cyclosporine in renal transplant recipients (BENEFIT study). Am J Transplant. 2010;10(3):535‐546. 20415897 10.1111/j.1600-6143.2009.03005.x

[5] Mathews DV , Wakwe WC , Kim SC , et al. Belatacept‐resistant rejection is associated with CD28. Am J Transplant. 2017;17(9):2285‐2299. 28502128 10.1111/ajt.14349 PMC5573634

[6] Tai X , Cowan M , Feigenbaum L , Singer A . CD28 costimulation of developing thymocytes induces Foxp3 expression and regulatory T cell differentiation independently of interleukin 2. Nat Immunol. 2005;6(2):152‐162. 15640801 10.1038/ni1160

[7] Malek TR , Yu A , Vincek V , Scibelli P , Kong L . CD4 regulatory T cells prevent lethal autoimmunity in IL‐2Rbeta‐deficient mice. Implications for the nonredundant function of IL‐2. Immunity. 2002;17(2):167‐178. 12196288 10.1016/s1074-7613(02)00367-9

[8] Furtado GC , Curotto de Lafaille MA , Kutchukhidze N , Lafaille JJ . Interleukin 2 signaling is required for CD4(+) regulatory T cell function. J Exp Med. 2002;196(6):851‐857. 12235217 10.1084/jem.20020190 PMC2194060

[9] Attridge K , Walker LS . Homeostasis and function of regulatory T cells (Tregs) *in vivo*: lessons from TCR‐transgenic Tregs. Immunol Rev. 2014;259(1):23‐39. 24712457 10.1111/imr.12165 PMC4237543

[10] Shevyrev D , Tereshchenko V . Treg heterogeneity, function, and homeostasis. Front Immunol. 2019;10:3100. 31993063 10.3389/fimmu.2019.03100 PMC6971100

[11] Dominguez‐Villar M , Hafler DA . Regulatory T cells in autoimmune disease. Nat Immunol. 2018;19(7):665‐673. 29925983 10.1038/s41590-018-0120-4 PMC7882196

[12] Wood KJ , Bushell A , Hester J . Regulatory immune cells in transplantation. Nat Rev Immunol. 2012;12(6):417‐430. 22627860 10.1038/nri3227

[13] Riella LV , Liu T , Yang J , et al. Deleterious effect of CTLA4‐Ig on a Treg‐dependent transplant model. Am J Transplant. 2012;12(4):846‐855. 22300534 10.1111/j.1600-6143.2011.03929.x

[14] Schwarz C , Unger L , Mahr B , et al. The immunosuppressive effect of ctla4 immunoglobulin is dependent on regulatory T cells at low but not high doses. Am J Transplant. 2016;16(12):3404‐3415. 27184870 10.1111/ajt.13872

[15] Matsuura A , Abe T , Yasuura K . Simplified mouse cervical heart transplantation using a cuff technique. Transplant. 1991;51(4):896‐898. 10.1097/00007890-199104000-00031 2014549

[16] Pilat N , Baranyi U , Klaus C , et al. Treg‐therapy allows mixed chimerism and transplantation tolerance without cytoreductive conditioning. Am J Transplant. 2010;10(4):751‐762. 20148810 10.1111/j.1600-6143.2010.03018.x PMC2856406

[17] Pilat N , Klaus C , Gattringer M , et al. Therapeutic efficacy of polyclonal tregs does not require rapamycin in a low‐dose irradiation bone marrow transplantation model. Transplant. 2011;92(3):280‐288. 10.1097/TP.0b013e3182241133 21697774

[18] Tourkova IL , Yurkovetsky ZR , Shurin MR , Shurin GV . Mechanisms of dendritic cell‐induced T cell proliferation in the primary MLR assay. Immunol Lett. 2001;78(2):75‐82. 11672590 10.1016/s0165-2478(01)00235-8

[19] Boyman O , Kovar M , Rubinstein MP , Surh CD , Sprent J . Selective stimulation of T cell subsets with antibody‐cytokine immune complexes. Science. 2006;311(5769):1924‐1927. 16484453 10.1126/science.1122927

[20] Mahr B , Unger L , Hock K , et al. IL‐2/alpha‐IL‐2 complex treatment cannot be substituted for the adoptive transfer of regulatory t cells to promote bone marrow engraftment. PLoS One. 2016;11(1):e0146245. 26731275 10.1371/journal.pone.0146245 PMC4701413

[21] Qureshi OS , Zheng Y , Nakamura K , et al. Trans‐endocytosis of CD80 and CD86: a molecular basis for the cell‐extrinsic function of CTLA‐4. Science. 2011;332(6029):600‐603. 21474713 10.1126/science.1202947 PMC3198051

[22] Noel G , Belghith M , Belanger B , Leduc C , Daniel C . Direct alloreactivity is more susceptible to regulation by natural regulatory T cells than indirect alloreactivity. J Immunol. 2013;190(7):3764‐3771. 23440413 10.4049/jimmunol.1200190

[23] Bluestone JA , Buckner JH , Fitch M , et al. Type 1 diabetes immunotherapy using polyclonal regulatory T cells. Sci Transl Med. 2015;7(315):315ra189. 10.1126/scitranslmed.aad4134 PMC4729454 26606968

[24] Wing K , Onishi Y , Prieto‐Martin P , et al. CTLA‐4 control over Foxp3+ regulatory T cell function. Science. 2008;322(5899):271‐275. 18845758 10.1126/science.1160062

[25] Klatzmann D , Abbas AK . The promise of low‐dose interleukin‐2 therapy for autoimmune and inflammatory diseases. Nat Rev Immunol. 2015;15(5):283‐294. 25882245 10.1038/nri3823

[26] Vaikunthanathan T , Safinia N , Boardman D , Lechler RI , Lombardi G . Regulatory T cells: tolerance induction in solid organ transplantation. Clin Exp Immunol. 2017;189(2):197‐210. 28422316 10.1111/cei.12978 PMC5508315

[27] Saadoun D , Rosenzwajg M , Joly F , et al. Regulatory T‐cell responses to low‐dose interleukin‐2 in HCV‐induced vasculitis. N Engl J Med. 2011;365(22):2067‐2077. 22129253 10.1056/NEJMoa1105143

[28] Koreth J , Matsuoka K‐I , Kim HT , et al. Interleukin‐2 and regulatory T cells in graft‐versus‐host disease. N Engl J Med. 2011;365(22):2055‐2066. 22129252 10.1056/NEJMoa1108188 PMC3727432

[29] Koreth J , Kim HT , Jones KT , et al. Efficacy, durability, and response predictors of low‐dose interleukin‐2 therapy for chronic graft‐versus‐host disease. Blood. 2016;128(1):130‐137. 27073224 10.1182/blood-2016-02-702852 PMC4937358

