# Supplementary figures and images for: In vivo Treg expansion under costimulation blockade targets early rejection and improves long‐term outcome
Source: Am J Transplant. 2021 Aug 23;21(11):3765–74. doi: 10.1111/ajt.16724 (PMC9292010; doi:10.1111/ajt.16724)

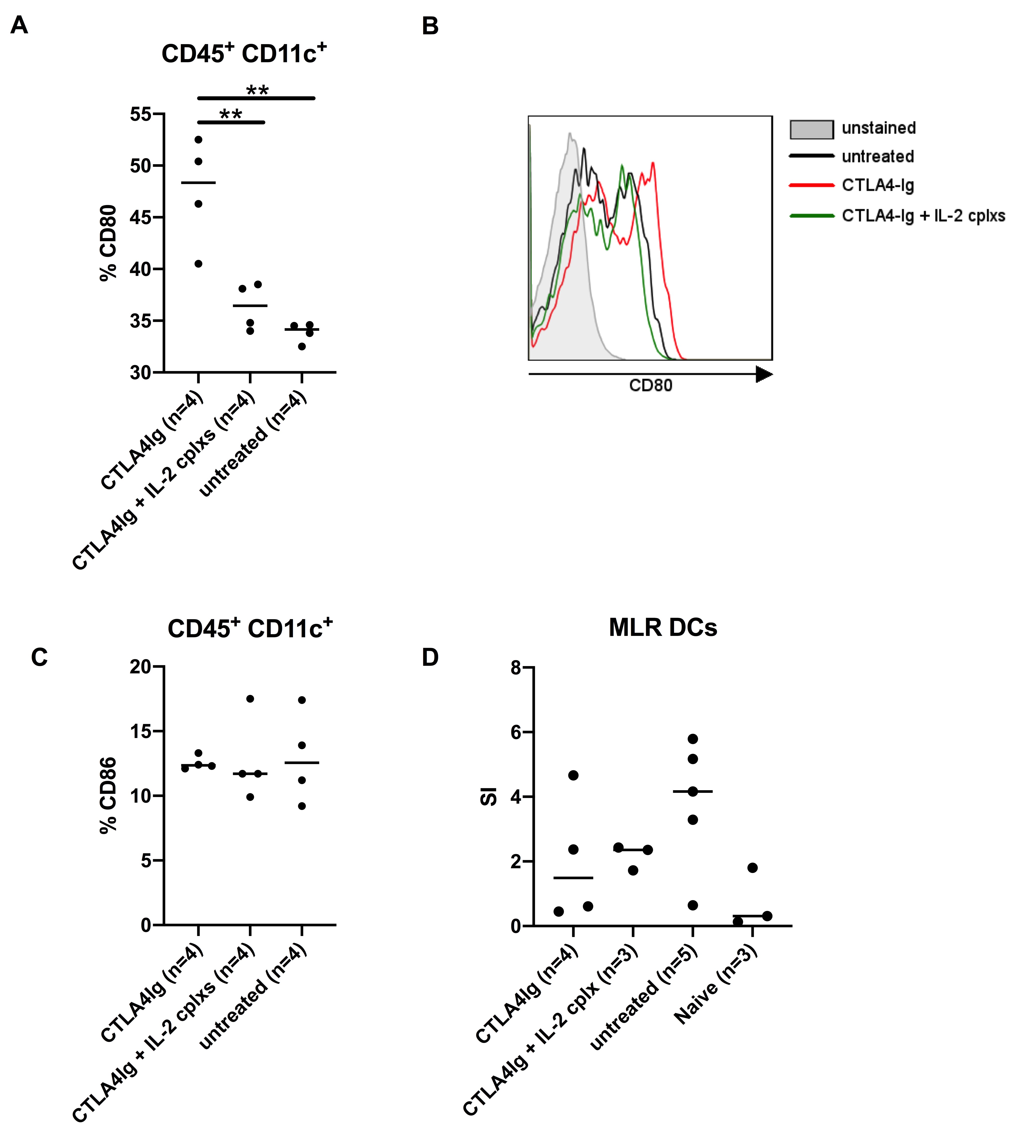

Supplement: Supplementary file 1 — Fig S1 [file AJT-21-3765-s001.jpg]
